# Cooler, stronger, smaller: improving thermoelectric cooling

**DOI:** 10.1093/nsr/nwae445

**Published:** 2024-12-04

**Authors:** Nagendra Singh Chauhan, Takao Mori

**Affiliations:** Research Center for Materials Nanoarchitectonics (MANA), National Institute for Materials Science (NIMS), Japan; Research Center for Materials Nanoarchitectonics (MANA), National Institute for Materials Science (NIMS), Japan; Graduate School of Pure and Applied Sciences, University of Tsukuba, Japan

**Figure 1.**
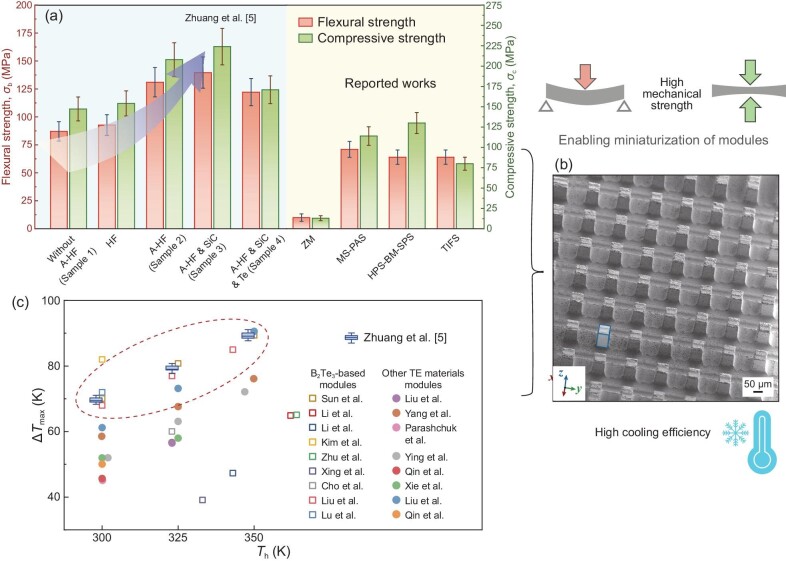
Schematic of process optimization for (Bi,Sb)_2_Te_3_-based nanocomposites, designed to enhance (a) mechanical strength and (c) cooling efficiency to fabricate micro-Peltier coolers having (b) micro cuboid pillar arrays, highlighting advancements in solid-state refrigeration. Adapted with permission from Ref. [5].

Bismuth telluride (Bi_2_Te_3_) alloys have long been the backbone of thermoelectric technology, driving breakthroughs in solid-state refrigeration and possible power generation for over 60 years [1–3]. With continuous advancements in both *n*-type (Bi_2_Te_3–_*_x_*Se*_x_*) and *p*-type (Bi*_x_*Sb_2–_*_x_*Te_3_) based compositions, Bi_2_Te_3_ continues to elevate thermal efficiency (*η*) and enhance the performance (coefficient of performance, COP) of cooling systems, reigning as the present champion material in commercial Peltier modules or thermoelectric coolers (TECs). Compact and reliable, TECs leverage the Peltier effect for precise micro-cooling in compact spaces, making them ideal for optoelectronics, wearable tech, and medical devices. The need for such TECs and relatively low temperature energy harvesting is intensifying, and also stimulating research into novel replacement materials such as Mg_3_(Sb,Bi)_2_ [4]. New research on Bi_2_Te_3_ by Zhuang *et al.* [5], published in *National Science Review*, reveals a multi-step process involving annealing, hot-forging and composition design to enhance both thermoelectric and mechanical performance of (Bi,Sb)_2_Te_3_ alloys by engineering atomic defects and optimizing nano-/micro-structures.

(Bi,Sb)_2_Te_3_ alloys are inherently brittle due to strong ionic/covalent bonding, low fracture strain, and anisotropic texturing, which boosts thermoelectric performance but compromises mechanical strength, creating a strength-performance trade-off [6–8]. The processed (Bi, Sb)_2_Te_3_ nanocomposites show remarkable mechanical enhancement, improved density and refined microstructures, yielding a peak *zT* of ∼1.5 with excellent processability, ideal for micro-TECs fabrication. Notably, flexural and compressive strengths increase by up to 50% (∼140 MPa) and 40% (∼224 MPa), respectively, in optimized samples—surpassing typical values obtained from conventional methods, involving ball milling, melt spinning and spark plasma sintering as shown in Fig. 1a. For instance, zone melting methods typically yield flexural and compressive strengths of only ∼10 MPa. Superior mechanical strength and high processability enables the fabrication of ultra-small (∼30 × 30 μm^2^) micro cuboid pillar arrays for TECs presented in Fig. 1b. Diced pillars, paired with commercial *n*-type Bi_2_Te_3_ legs, support scalable production of micro-PCs with outstanding performance, achieving peak cooling of Δ*T*_max_ ∼ 89.3 K and a COP ∼ 6.6, surpassing conventional Bi_2_Te_3_-based devices as displayed in Fig. 1c.

Hot forging creates dense, high-strength microstructures with minimal micropores and increased dislocations [8,9], while nano SiC particles enhance strength through strain and dispersion effects, lowering thermal conductivity simultaneously. Adding excess Te reduces vacancy concentration by creating nanoscale lattice distortions and dense dislocations, which improves carrier concentration and mobility, thereby enhancing thermoelectric performance [1–3]. The process optimizations of annealing-hot forging process and nano SiC-Te incorporation in (Bi,Sb)_2_Te_3_ nanocomposites enhances electrical conductivity, weighted mobility, and power factor, boosting *zT* through synergistically reduced lattice thermal conductivity, realizing *η ∼*7.5% at *ΔT* = 225 K.

In summary, Zhuang *et al*. [5] demonstrates scalable (Bi,Sb)_2_Te_3_ micro-TECs, measuring just 2 × 2 mm^2^ to achieve impressive cooling (Δ*T*_max_ up to 89.3 K, *zT* ∼1.50 at a *T_h_* ∼ 348 K) and enhanced mechanical strength, which is promising for efficient micro-TECs in solid-state refrigeration. Seiko previously fabricated thermoelectric watches utilizing sub-millimeter sized Bi_2_Te_3_-based thermoelectric generator (TEG) legs [10]. As further general issues for development of various thermoelectric applications, inexpensive thermoelectric module fabrication methods remain an important challenge. Advancements in thermoelectric cooling towards more efficient, precise, compact and intelligent thermal management solutions, is poised to play a vital role in next-generation high-performance micro- and opto-electronic devices.
